# Cost-effectiveness of spinal manipulation, exercise, and self-management for spinal pain using an individual participant data meta-analysis approach: a study protocol

**DOI:** 10.1186/s12998-018-0216-9

**Published:** 2018-11-13

**Authors:** Brent Leininger, Gert Bronfort, Roni Evans, James Hodges, Karen Kuntz, John A. Nyman

**Affiliations:** 1 Integrative Health & Wellbeing Research Program, Earl E. Bakken Center for Spirituality & Healing, University of Minnesota, 420 Delaware St SE, Minneapolis, MN 55455 USA; 2 Division of Biostatistics, School of Public Health, University of Minnesota, 420 Delaware St SE, Minneapolis, MN 55455 USA; 3 Department of Health Policy and Management, University of Minnesota, 420 Delaware St SE, Minneapolis, MN 55455 USA

**Keywords:** Cost-effectiveness, Back pain, Neck pain, Exercise, Spinal manipulation, Self-care, Randomized clinical trial

## Abstract

**Background:**

Spinal pain is a common and disabling condition with considerable socioeconomic burden. Spine pain management in the United States has gathered increased scrutiny amidst concerns of overutilization of costly and potentially harmful interventions and diagnostic tests. Conservative interventions such as spinal manipulation, exercise and self-management may provide value for the care of spinal pain, but little is known regarding the cost-effectiveness of these interventions in the U.S. Our primary objective for this project is to estimate the incremental cost-effectiveness of spinal manipulation, exercise therapy, and self-management for spinal pain using an individual patient data meta-analysis approach.

**Methods/design:**

We will estimate the incremental cost-effectiveness of spinal manipulation, exercise therapy, and self-management using cost and clinical outcome data collected in eight randomized clinical trials performed in the U.S. Cost-effectiveness will be assessed from both societal and healthcare perspectives using QALYs, pain intensity, and disability as effectiveness measures. The eight randomized clinical trials used similar methods and included different combinations of spinal manipulation, exercise therapy, or self-management for spinal pain. They also collected similar clinical outcome, healthcare utilization, and work productivity data. A two-stage approach to individual patient data meta-analysis will be conducted.

**Discussion:**

This project capitalizes on a unique opportunity to combine clinical and economic data collected in a several clinical trials that used similar methods. The findings will provide important information on the value of spinal manipulation, exercise therapy, and self-management for spinal pain management in the U.S.

## Background

Chronic pain is a major public health problem, affecting more adults in the United States than heart disease, diabetes, and cancer combined [1]. Low back and neck pain are the first and third most common chronic pain conditions in U.S. adults [2] with nearly one of three Americans experiencing chronic neck or low back pain in their lifetime [3]. While the prevalence of spinal pain has been stable over the past two decades, the global burden (measured by disability adjusted life years) has increased 42% due to aging and population growth [4, 5]. Roughly 25% of U.S. adults with spine pain report limitations with physical function, 11% report limitations with social function, and 19% report limitations with work, school or household activities [6]. With low back pain ranked first and neck pain fourth in disability worldwide, spine pain has become a burdensome health condition with considerable public health consequences [4, 5].

The economic impact of spinal pain and associated comorbidities is also substantial. An estimated $88.1 billion in healthcare expenditures (~ 4% of total healthcare spending) was directed towards low back and neck pain within the U.S. in 2013 [7]. Diabetes and ischemic heart disease are the only conditions in the U.S. with higher levels of healthcare spending [7]. While direct expenditures for spinal pain are large, total healthcare expenditures are even greater due to comorbidities (such as depression) [3]. When total healthcare costs are considered, for example care of spinal pain plus associated comorbid conditions, expenditures increase to 9% of total U.S. healthcare costs (2005 data) [6]. In addition, reduced work productivity accounts for a large proportion of the financial burden in individuals with back or neck pain. Lost productivity costs for back pain were estimated at $19.8 billion per year in 2002, with reduced productivity while still at work (i.e. presenteeism) accounting for nearly 70% of total lost productivity costs [8].

Spine pain management has gathered increased scrutiny amidst concerns about overutilization of costly and potentially harmful interventions and diagnostic tests. Over the past decade the number of epidural injections, opioid prescriptions, and spinal surgeries for back pain has more than doubled with little positive impact on patient outcomes [9–13]. Annual healthcare expenditures for individuals with spinal pain increased by 95% from 1999 to 2008 largely due to increases in medical specialist expenditures [14]. From 1996 to 2013, the U.S. spent an additional $57.2 billion dollars per year on the management of spinal pain, which represents one of the larger increases in healthcare spending for any condition [7]. While more conservative and potentially less costly alternatives are available to treat spinal pain, including spinal manipulation therapy (SMT), exercise therapy, and self-management, they are often underutilized. An analysis of administrative data from across the U.S. found low back pain patients are more likely to receive opioids (41%) than visit a chiropractor (39%) or physical therapist (34%) [15].

Complementary and integrative interventions may reduce the clinical and cost burden of spine pain. Recent data from the U.S. Medical Expenditures Panel Survey suggests complementary and integrative therapies, including SMT, reduce healthcare expenditures for spinal pain conditions; however, the cost-effectiveness of SMT within U.S. healthcare settings has not received much attention [16–18]. Given the increasing financial and societal burden of spinal pain, and concerns surrounding current management strategies, robust cost-effectiveness analyses (CEA) of SMT and other complementary and integrative treatments for spine pain are much needed [18–21].

### Study objectives

Our primary objective is to estimate the incremental cost-effectiveness of spinal manipulation, exercise therapy, and self-management for spinal pain from both societal and healthcare perspectives using quality-adjusted life years (QALYs), pain intensity, and disability as effectiveness measures. We will analyze cost and clinical outcome data collected as part of eight randomized clinical trials performed in the U.S. using an individual patient data meta-analysis (IPDMA) approach. The eight randomized clinical trials used similar methods, collected similar clinical outcome, healthcare utilization, and work productivity data, and included different combinations of SMT, exercise therapy, or self-management for spinal pain.

## Methods

We will use an IPDMA approach to evaluate costs and effects of spinal manipulation, exercise therapy, and self-management (i.e. home exercise & advice) for spinal pain using data from eight randomized clinical trials including a total of 1891 participants (Table 1). Each of the included trials obtained written consent from participants who were 18 years of age or older, and written patient assent and parent consent from participants 12–17 years of age. Six of the clinical trials were funded by the U.S. Department of Health and Human Services Health Resources and Services Administration [22–27] and one was funded by the National Institute of Health’s National Center for Complementary and Integrative Health [28]. Seven of the trials are registered on clinicaltrials.gov (one trial [29] was initiated prior to its existence).

**Table 1:** Clinical trial populations and interventions

	Population			Interventions		
	Condition	Sample	Age	Group 1	Group 2	Group 3
Pre-dates CT.gov [29]	Chronic neck pain	191	20–65	SMT + ET	ET	SMT
NCT00269360 [22]	Chronic neck pain	270	18–65	SMT + ET	ET	SC
NCT00269308 [23]	Chronic neck pain	241	65+	SMT + SC	ET + SC	SC
NCT00029770 [28]	Acute neck pain	272	18–65	SMT	SC	Medication
NCT00269347 [24]	Chronic low back pain	301	18–65	SMT	ET	SC
NCT00269321 [25]	Chronic low back pain	240	65+	SMT + SC	ET + SC	SC
NCT00494065 [26]	Chronic back-related leg pain	192	21+	SMT + SC	SC	–
NCT01096628 [27]	Chronic low back pain	184	12–18	SMT + ET	ET	–

*CNP* chronic neck pain, *ANP* acute neck pain, *CLBP* chronic low back pain, *BRLP* back related leg pain, *SMT* spinal manipulation therapy, *ET* exercise therapy, *SC* self-care

### Settings and participants

All of the clinical trials were performed within a university-affiliated research clinic in the Minneapolis, MN metropolitan region. Six of the clinical trials were performed exclusively in MN [22–25, 28, 29] and two were multi-center studies with additional sites in Portland, OR [27] or Davenport, IA [26]. Participants had commonly recognized sub-groups of spinal pain including acute or chronic neck pain, chronic low back pain, and back-related leg pain (Table 1). Five of the clinical trials included adults (18–65 years), two trials included older adults (65 years and older), and one trial focused on adolescents (12–18 years). All eight trials recruited participants from the general population primarily through mass mailings. Other recruitment strategies included advertisement in newspapers, social media, television, radio, and community posters.

### Inclusion/exclusion criteria

In all 8 of the clinical trials, participants were required to have self-reported severity of spinal pain ≥3/10 for inclusion. Other inclusion criteria common to the 8 clinical trials included a stable medication plan and no ongoing spinal treatment prior to enrollment. Common exclusion criteria included current or pending litigation, inability to read and comprehend English, substance abuse, history of surgical spinal fusion, progressive neurological deficits, or contraindications to study treatments.

### Interventions

Spinal manipulation was included as an intervention in all eight trials. Supervised exercise therapy was provided in six trials and a self-management intervention focusing on home exercises and advice was also provided in six of the trials.

*Spinal manipulation therapy (SMT)* was delivered by licensed chiropractors over an 11–12 week intervention period in all eight trials. The treating chiropractor determined the frequency of SMT visits in six of the eight studies. The mean frequency of SMT visits ranged from 10 to 20 across the eight trials with most trials reporting a mean visit frequency between 15 and 20. SMT consisted of high velocity, low amplitude manipulation, with an option to use low velocity, variable amplitude mobilization as indicated. Brief soft tissue work (up to 5 minutes) and heat therapy were allowed to facilitate the manual treatment, if necessary, which is typical in clinical practice.

*Supervised exercise therapy* was delivered by licensed chiropractors, physical therapists or exercise therapists over an 11–12 week period in six trials. Five studies [22–25, 29] included 20 one-hour visits and one trial [27] included 8 to16, 45-min visits of one-on-one supervised exercise therapy [25, 29]. Participants completed a combination of stretching and strengthening exercises emphasizing a high number of repetitions with progressions in challenge and/or resistance over time. Exercises were tailored for each participant’s abilities and could include the use of labile surfaces in addition to balance and coordination exercises. Participants also completed a light aerobic warm-up (up to 10 min) in all of the clinical trials.

*Self-management* was delivered by licensed chiropractors or exercise therapists in six trials [22–26, 28]. Participants attended two to four one-hour visits where they were given information on basic spinal anatomy and physiology, their prognosis, self-care advice (such as heat or icing instructions), postural and ergonomic advice, and home exercise instruction. Home exercises typically included a combination of self-mobilization, stretching and strengthening exercises specific to the individual’s condition and ability.

### Perspective, Time Horizon & Discount Rate

We will adopt a societal perspective for the primary analysis including all healthcare costs regardless of payer (third-party insurers, patient out-of-pocket costs) in addition to time and transportation costs associated with healthcare use and lost productivity costs for both paid and unpaid labor related to spinal pain. We will exclude future earnings and consumption costs since interventions for spinal pain are not expected to impact mortality. In addition to the societal perspective, we will adopt a healthcare perspective including only healthcare costs [30]. A summary of resources included in the healthcare and societal perspectives are provided in Table 2. We will not adopt a patient perspective as a secondary analysis, as patient level costs (copays, co-insurance) vary considerably by health insurance plan in the U.S. and we do not have access to this data for trial participants. All eight clinical trials collected clinical outcome and healthcare utilization data for 1 year following randomization, which will therefore be the time horizon for the cost-effectiveness evaluation. No discounting (diminishing future costs and health effects to represent present value) will be applied.

**Table 2:** Cost components included in the societal and healthcare perspectives

Cost component	Perspective	
	Healthcare	Societal
*Formal healthcare sector*		
Paid for by third-party payers	X	X
Paid for by patients	X	X
*Informal healthcare sector*		
Patient time	–	X
Transportation costs	–	X
*Non-healthcare sector*		
Productivity costs (paid and unpaid labor)	–	X

### Outcomes

In all trials, clinical and cost outcomes were collected by self-report at multiple time points over a 1 year period with similar timing (4, 12, 26, and 52 weeks). All eight trials collected clinical outcomes including pain, disability, quality of life, and work absenteeism (Table 3).

**Table 3:** Clinical trial outcomes

Condition (ClinicalTrials.gov ID)	Pain	QALY	Disability	Medication use	Healthcare Utilization (Questionnaire)	Healthcare Utilization (Interview)	Productivity Costs
Pre-dates CT.gov [29]	X	SF6D	NDI	X	X	–	X
NCT00269360 [22]	X	SF6D/EQ5D	NDI	X	X	X	X
NCT00269308 [23]	X	SF6D/EQ5D	NDI	X	X	X	X
NCT00029770 [28]	X	SF6D/EQ5D	NDI	X	X	–	X
NCT00269347 [24]	X	SF6D/EQ5D	RM	X	X	X	X
NCT00269321 [25]	X	SF6D/EQ5D	RM	X	X	X	X
NCT00494065 [26]	X	SF6D/EQ5D	RM	X	X	X	X
NCT01096628 [27]	X	PedsQL	RM	X	X	–	–

*QALY* Quality Adjusted Life Year, *NDI* Neck Disability Index, *RM* Roland Morris Back Disability

#### Clinical outcomes

QALYs (a metric combining quality and quantity of life) will be constructed for all eight trials, using the SF6D, which was collected in seven trials, and the EQ5D-3 L, which was collected in six trials. The SF6D is derived from 11 items within the Medical Outcomes Study Short Form 36-item Health Survey (SF-36) and includes six dimensions of health, each with 4–6 different levels [31]. We will use U.S. preferences for individual SF6D health states obtained via discrete choice experimentation, which are similar to UK values elicited with standard gamble methods [32]. In addition, QALYs will be estimated using the EuroQol 5D-3 L (EQ5D-3 L) as a sensitivity analysis. The EQ5D-3 L measures health across five dimensions, each with three possible levels. Preferences for EQ5D-3 L health states will be determined using published values from a representative sample of the U.S. population elicited by time trade-off methods [33]. Finally, one study of adolescents [27] assessed health related quality of life using the pediatric quality of life inventory (PedsQL) which has recently been mapped to the EQ5D in a UK adolescent population [34].

Self-reported pain intensity was measured using the 11-box numerical rating scale (NRS) and was the primary outcome in each of the eight trials. The NRS is a reliable and valid outcome measure for individuals with spinal pain and is recommended as a core outcome domain by both the Initiative on Methods, Measurement, and Pain Assessment in Clinical Trials group and the NIH task force on research standards for chronic low back pain [35, 36].

Disability was measured with reliable and valid measures commonly used in spine pain research: the Neck Disability Index (four trials) [37, 38] and Roland-Morris Disability Questionnaire (four trials) [39]. The CEA using disability as the effectiveness outcome will use standardized mean differences due to the variation in disability measures. Standardized mean differences provide a uniform scale for meta-analysis of outcomes measured with different psychometric scales. The standardized mean difference reports the size of the treatment effect relative to the amount of variability between participants in the same treatment group within each study [40]. A standardized mean difference of one equates to a treatment effect that is the size of one standard deviation across studies.

#### Cost outcomes

##### Direct healthcare costs

Healthcare utilization outcomes collected include the number of provider visits by specialty, types of services provided, and medication use. The number of provider visits and medication use were collected using standardized self-report questionnaires in all eight trials, and more detailed information regarding the types of services provided (e.g. MRI, injections) was collected by phone interviews in five of the trials. A list of procedures and corresponding Current Procedural Terminology (CPT)/ Healthcare Common Procedure Coding System (HCPCS) codes will be compiled and unit costs for each procedure will be determined using Medicare’s national allowable payment. Unit costs for non-covered services under Medicare (such as acupuncture) will be determined using Medicare’s published relative value unit for the corresponding CPT code. Unit costs for medication will be determined using the average cost per prescription day from Medicare’s prescription drug profiles public use file. All unit cost estimates will be converted to a recent common price year in U.S. dollars using the Centers for Medicare and Medicaid Services Personal Health Care Expenditure deflator to account for inflation [30].

##### Productivity costs

A human capital approach including lost productivity costs for both paid and unpaid labor (such as retirees or homemakers) will be used [41]. Lost work productivity due to absenteeism was collected using a modified question from the U.S. National Health Interview Survey (seven trials) [42]. Participants reported the number of days in the past month they were unable to carry out their daily work (in or away from home) due to spine pain. We will value each day as 8 hours of reduced productivity using age specific U.S. national pre-tax median hourly wage rates plus fringe benefits [30]. We will report productivity costs for paid labor, unpaid labor and their combined total.

##### Time & Transportation Costs

Time and transportation costs associated with healthcare utilization will be included using an opportunity cost approach (valuing resources according to their best alternative use). A standardized time unit for each procedure will be multiplied by the age specific U.S. national post-tax median wage rate plus fringe benefits for each participant. Healthcare related transportation costs will be estimated using average distance and transportation time estimates for medical care in the U.S. as reported in the National Household Travel Survey [43]. Transportation time will be valued using age-specific national post-tax median wage rates plus fringe benefits [30]. The U.S. Internal Revenue Service’s standard mileage deduction rate for operating an automobile will be used to value transportation costs.

### Analysis

#### Effectiveness analyses

Clinical outcomes will be analyzed using hierarchical linear mixed models to account for correlation within subjects due to repeated measures. Time weighted averages using linear interpolation will be used to determine mean clinical outcomes over the 1 year time horizon. Baseline clinical outcomes (QALYs, pain, and disability) will be included as covariates.

#### Cost outcomes analyses

Cost data for healthcare and medication use, time and transportation, and lost productivity will be analyzed using generalized estimating equations specifying an appropriate distribution (such as Poisson, negative binomial, or gamma) and link to model mean costs over the 1 year time horizon [44]. Cost distributions are frequently skewed with a heavy tail as a small number of participants are responsible for a large proportion of cost. We will account for this by bootstrapping cost and outcome data using 5000 bootstrap samples taken with replacement with the trial participant as the unit of observation.

#### Cost-effectiveness analyses

We will rank treatments by mean outcome and determine the incremental cost-effectiveness ratio (ICER) by dividing incremental costs by incremental effects. We will not calculate ICERs for treatments which are dominated (more expensive, less effective); however, we will report uncertainty of cost and effect differences for dominated interventions [45]. Bias-corrected bootstrap confidence intervals will be calculated and the bootstrapped cost-effect pairs will be plotted on the cost-effectiveness plane to graphically display uncertainty surrounding the ICER [46]. We will use cost-effectiveness acceptability curves (graphical display of uncertainty that an intervention is cost-effective at different willingness to pay thresholds for 1 year of perfect health) to determine the probability each treatment is cost-effective based on a range of recommended willingness to pay thresholds for a QALY within the U.S [47]. Additionally, we will use net monetary benefit analyses to present confidence intervals over a large range of willingness to pay thresholds (the amount of money a decision maker would be willing to pay for 1 year of full health given a fixed budget) [48].

#### Missing data analyses

The pattern of missing clinical outcome and cost data will be assessed to determine the appropriate imputation strategy and sensitivity analysis. If data are missing at random (differences between missing and observed data are related only to other observed data), multiple imputation and likelihood based estimation techniques such as linear mixed models will be used [49, 50]. If there is suspicion that data are not missing at random, sensitivity analyses will be performed by imputing data accounting for missingness explanations (such as refusal of assigned treatment or dropout due to poor treatment response).

### Individual participant data meta-analysis (IPDMA)

We will use a two-stage approach for IPDMA and will follow recommended guidelines for standard and IPDMA analyses [51–54].

Stage One: First, for each perspective (societal, healthcare) and comparison (e.g. SMT vs self-management), we will identify trials for possible meta-analysis. Next, we will determine individual trial estimates for differences in effectiveness, costs, and incremental cost-effectiveness.

Stage Two: We will combine studies using random effects models [54] to produce pooled cost-effectiveness estimates. Prior to pooling, we will visually inspect individual trial estimates of effectiveness and costs using forest plots and determine the amount of statistical heterogeneity using the I^2^ statistic. If no more than minimal heterogeneity is detected for both estimates of effectiveness and costs (I^2^ < 50%, with consistent direction and magnitude of estimates), we will report the pooled estimates. It is possible we will encounter more than minimal heterogeneity when attempting to combine data from multiple spinal pain trials. If heterogeneity prevents statistical pooling and cannot be explained by a priori defined sub-group analyses, we will report the individual trial cost-effectiveness results.

Regardless of our ability to pool, we will perform a limited number of a priori determined subgroup analyses, which are especially relevant for decisions in clinical practice [53, 55]. This may explain heterogeneity (if it exists) identified in stage two of the IPDMA. We will determine if differences in cost-effectiveness exist due to 1) age; 2) pain location (neck, back, and leg pain); 3) duration of pain (acute vs chronic); and 4) type of comparison (head to head vs add-on comparison; e.g. SMT+ supervised exercise vs supervised exercise alone).

## Discussion

Combined analyses of economic data are rarely possible due to differences in resource utilization outcomes, costs and healthcare settings [56, 57]. Additionally, individual clinical trials rarely include a sufficient number of participants to detect important differences in economic outcomes. This project represents a unique opportunity to potentially combine clinical and economic data collected in eight randomized clinical trials using an IPDMA approach. This will provide more precise estimates of the cost-effectiveness of spinal manipulation, exercise therapy, and self-management compared to analysis of the individual trials. Further, an IPDMA approach has many advantages over traditional meta-analysis including the ability to conduct standardized within-study analyses, account for missing data at the individual level, and investigate potential sub-group effects at the participant level which may account for heterogeneity in estimates across studies [52].

While systematic reviews have found promising evidence of the cost-effectiveness of SMT for spinal pain, particularly when productivity costs are considered, [58–62] the original studies have limitations that draw attention to the need for further high quality CEAs [18, 20]. Few existing CEAs have adopted both societal (including lost productivity costs) and healthcare perspectives [59, 61, 62] to facilitate the applicability of findings to multiple audiences (including policy makers and health-care systems) [30]. Additionally, high rates of missing cost and QALY data are common among the existing studies. This project will address these issues by using an IPDMA approach to combine data from multiple, comparable RCTs with high follow up rates, and will report cost-effectiveness estimates from both societal and healthcare perspectives. Limitations of our approach include relying on participant self-report for non-study related healthcare use, the absence of costs for informal caregiver time due to spinal pain within the societal perspective, and the potential that interventions within the trials were protocolized relative to real world application.

The generalizability of existing cost-effectiveness studies to the U.S. healthcare system is also a concern [18, 20, 21]. A limited number of studies have been conducted in the U.S [62, 63]. Further, few studies have assessed SMT delivered by chiropractors [62, 64], the profession responsible for providing the majority of SMT services in the U.S [65]. Cost-effectiveness research on SMT from European healthcare settings does not readily transfer to the U.S. where healthcare costs are much higher [66, 67]. For this project, all of the trials were performed within the U.S. and assessed SMT delivered by chiropractors. This project will fill an important gap in providing new information regarding the value SMT, exercise therapy, and self-management for managing spinal pain within the U.S. healthcare system. Comparisons to U.S. based cost-effectiveness estimates for other common spinal pain treatments will be made when possible to provide context regarding the relative value of these treatments.

## Abbreviations

CEA: Cost-effectiveness analysis
CPT: Current Procedural Terminology
HCPCS: Healthcare Common Procedure Coding System
IPDMA: Individual participant data meta-analysis
NRS: Numerical rating scale
PedsQL: Pediatric quality of life inventory
QALY: Quality-adjusted life year
RCT: Randomized clinical trial
SMT: Spinal manipulation therapy
